# Low-frequency STN-DBS provides acute gait improvements in Parkinson’s disease: a double-blinded randomised cross-over feasibility trial

**DOI:** 10.1186/s12984-021-00921-4

**Published:** 2021-08-10

**Authors:** Zachary J. Conway, Peter A. Silburn, Thushara Perera, Karen O’Maley, Michael H. Cole

**Affiliations:** 1 School of Behavioural and Health Sciences, Australian Catholic University, P.O. Box 456, Brisbane, QLD 4014 Australia; 2 Asia-Pacific Centre for Neuromodulation, Queensland Brain Institute, The University of Queensland, Brisbane, QLD Australia; 3 Neurosciences Queensland, Brisbane, QLD Australia; 4 The Bionics Institute, East Melbourne, VIC Australia; 5 Department of Medical Bionics, The University of Melbourne, Parkville, VIC Australia; 6 Development and Disability over the Lifespan Program, Healthy Brain and Mind Research Centre, Australian Catholic University, Brisbane, Australia

**Keywords:** UPDRS, Walk, Rhythmic, Sensor, Balance

## Abstract

**Background:**

Some people with Parkinson’s disease (PD) report poorer dynamic postural stability following high-frequency deep brain stimulation of the subthalamic nucleus (STN-DBS), which may contribute to an increased falls risk. However, some studies have shown low-frequency (60 Hz) STN-DBS improves clinical measures of postural stability, potentially providing support for this treatment. This double-blind randomised crossover study aimed to investigate the effects of low-frequency STN-DBS compared to high-frequency stimulation on objective measures of gait rhythmicity in people with PD.

**Methods:**

During high- and low-frequency STN-DBS and while off-medication, participants completed assessments of symptom severity and walking (e.g., Timed Up-and-Go). During comfortable walking, the harmonic ratio, an objective measures of gait rhythmicity, was derived from head- and trunk-mounted accelerometers to provide insight in dynamic postural stability. Lower harmonic ratios represent less rhythmic walking and have discriminated people with PD who experience falls. Linear mixed model analyses were performed on fourteen participants.

**Results:**

Low-frequency STN-DBS significantly improved medial–lateral and vertical trunk rhythmicity compared to high-frequency. Improvements were independent of electrode location and total electrical energy delivered. No differences were noted between stimulation conditions for temporal gait measures, clinical mobility measures, motor symptom severity or the presence of gait retropulsion.

**Conclusions:**

This study provides evidence for the acute benefits of low-frequency stimulation for gait outcomes in STN-DBS PD patients, independent of electrode location. However, the perceived benefits of this therapy may be diminished for people who experienced significant tremor pre-operatively, as lower frequencies may cause these symptoms to re-emerge.

*Trial registration*: This study was prospectively registered with the Australian and New Zealand Clinical Trials Registry on 5 June 2018 (ACTRN12618000944235).

**Supplementary Information:**

The online version contains supplementary material available at 10.1186/s12984-021-00921-4.

## Introduction

Deep brain stimulation (DBS) of the subthalamic nucleus (STN-DBS) has become a common procedure for improving symptoms of Parkinson’s disease (PD), such as resting tremor and limb stiffness, that are refractory to pharmacological treatments [1]. However, postural instability, a symptom strongly associated with falling in those with PD [2], declines following STN-DBS [3] and subsequently has been considered a contributing factor to the increased falls rate reported for those who are more than one year post-surgery [4]. Such research has led to suggestions that high-frequency STN-DBS stimulation may be inadequate for managing symptoms of postural instability in PD populations. Due to this potential shortcoming of the therapy, people with PD who are receiving STN-DBS would likely exhibit an increased falls risk following surgery [5].

In response to the documented increase in postural instability, gait disability, and the subsequent falls risk, researchers have investigated whether altering stimulation parameters (e.g. voltage amplitude or stimulation frequency) improves the post-operative management of such symptoms. Previous studies have found low-frequency stimulation (60–80 Hz) improves axial motor symptoms (e.g. postural stability) with no significant adverse effects on the management of limb tremor [6, 7]. Whilst the use of low-frequency STN-DBS seems beneficial for improving axial symptoms when compared to high-frequency stimulation [8], the exact therapeutic mechanism for this improvement remains unconfirmed. Furthermore, to date, the reported changes in motor symptoms in response to low-frequency STN-DBS stimulation strategies have been based almost exclusively on well-established, though often subjective, clinical scales or spatial–temporal measures [9]. However, recent research involving optimally-medicated people with PD has provided evidence to suggest that inexpensive and unobtrusive wearable sensors can provide important insight into changes in balance [10] and gait [11–13] in this population; potentially adding value to current clinical practices.

Of the research that has utilized acceleration-derived measures in optimally-medicated people with PD, the harmonic ratio is the most commonly reported measure of dynamic postural stability [14]. The harmonic ratio uses gait-related accelerations to provide a unique measure of one’s gait rhythmicity and dynamic postural stability [11, 15–20]. Less rhythmic gait patterns are exhibited by people who have greater difficulty adjusting to the small postural challenges often associated with walking. This difficulty is reflected by lower harmonic ratios, with research showing that lower harmonic ratios discriminate people with PD who experience falls from those who do not [11, 20]. Although this objective measure has been extensively used in the literature [14], to date, no study has used the harmonic ratio to understand gait-related changes in STN-DBS PD patients or the effect of low-frequency stimulation on dynamic postural stability. This feasibility study employed a double-blind randomised crossover design to investigate the effects of low-frequency STN-DBS on objective measures of gait rhythmicity in people with PD. It was hypothesized that low-frequency stimulation would significantly improve gait rhythmicity (higher harmonic ratios) compared to the usual high-frequency stimulation setting.

## Methods

### Participants

Participants were randomly recruited from a neurology clinic and local support groups (Table 1) and were accepted into the study if they were; clinically-diagnosed with idiopathic PD; aged between 50 and 75 years; had undergone bilateral STN-DBS surgery no less than 12-months earlier; independently living within the community; able to stand and ambulate without assistance; free of any significant musculoskeletal or medical conditions (other than PD); not taking medications that would adversely affect their balance; and free of any signs of dementia (Standardized Mini-Mental State Examination score < 24) [21]. This study was approved by the Australian Catholic University’s Human Research Ethics Committee (2017-155H) and volunteers provided written informed consent prior to participation. Given the lack of data concerning gait rhythmicity compared to temporal gait measures for people with PD following STN-DBS PD, gait rhythmicity measures collected for optimally-medicated people with PD were used to derive an a priori sample size estimate. It was determined a minimum of 12 participants was required to detect differences between high- and low-frequency stimulation (Effect Size ≥ 0.82 Power = 0.8, p = 0.05). This trial was registered on 5/06/2018 (ACTRN12618000944235).

**Table 1:** Demographic information and disease-specific characteristics for the STN-DBS PD patients

	*n* = *14*
*Demographics*	
Gender (male)^a^	12.0 (85.7)
Age (years)	69.6 (7.5)
Height (m)	1.8 (0.1)
Mass (kg)	81.3 (15.1)
*Falls history and fear of falls*	
Retrospective faller^a^	7.0 (50.0)
ABC-6 (max. score = 100)	53.8 (23.6)
*Neurological examination*	
Disease duration (years)	12.0 (6.2)
MDS-UPDRS III	32.7 (10.7)
Experience freezing of gait^a^	5.0 (35.7)
New freezing of gait questionnaire	19.8 (5.8)
8-Item Parkinson’s disease questionnaire	27.7 (15.9)
No PD medications^a^	7.0 (50.0)
Levodopa dose (mg/day)	271.5 (115.0)
Dopamine agonists^a^	4.0 (28.6)
Monoamine oxidase type B inhibitors^a^	0.0 (0.0)
Catechol-o-methyl transferase inhibitors^a^	0.0 (0.0)
*DBS information*	
Time since STN-DBS (years)	4.0 (2.4)
Euclidean distance^b^	2.36 (0.32–5.17)
X distance (negative = medial)^b^	− 0.90 (− 3.17–1.72)
Y distance (negative = posterior)^b^	− 0.35 (− 1.80–3.45)
Z distance (negative = inferior)^b^	− 0.74 (− 4.33–2.68)

Data represent mean (± 1 standard deviation), absolute numbers (percentage of sample)^a^ or mean (range)^b^

*ABC-6* 6-item Activities-specific Balance Confidence scale, *MDS-UPDRS III* motor subscale of the Movement Disorders Society-Sponsored Revision of the Unified Parkinson’s Disease Rating Scale

### STN-DBS interventions

Following overnight withdrawal of antiparkinsonian medications (≥ 12 h), participants attended a testing session held in a dedicated research space within a neurology clinic. On arrival, a registered nurse who specialised in the management of those with STN-DBS determined the DBS electrode impedance and calculated the total electrical energy delivered (TEED) for the participants’ chronic stimulation settings [22]. Using a one-to-one allocation ratio, the DBS nurse, informed by a computer-generated randomisation sequence, programmed the STN-DBS electrodes to one of two therapeutic conditions; (i) high-frequency; or (ii) low-frequency stimulation. Specifically, the high-frequency condition involved the STN-DBS electrodes being bilaterally active at the high-frequency stimulation (> 100 Hz) that the participants routinely received. Low-frequency stimulation involved electrodes being bilaterally set to a lower frequency (60 Hz) with the voltage increased to maintain the TEED consistent with the participant’s high-frequency (chronic) stimulation setting. A one-hour wash-in period was enforced between high-frequency and low-frequency conditions to limit the risk of any carry-over effects [23]. To limit the risk of bias, only the DBS nurse was aware of the STN-DBS settings; hence, both the participant and the researchers administering the assessments were blinded.

### Procedures

Prior to attending the session, participants completed a series of questionnaires to establish their medical history and medication use, while freezing of gait history and balance confidence were evaluated using the revised Freezing of Gait questionnaire [24] and the 6-item Activities-specific Balance Confidence scale [25], respectively. During each therapeutic condition, symptom severity was assessed by a movement scientist using part three (motor sub-section) of the Movement Disorders Society-Sponsored Revision of the Unified Parkinson’s Disease Rating Scale (MDS-UPDRS III). The total score for this sub-section and the result for item 12 (retropulsion test) were both reported, with higher scores representing greater symptom severity and poorer postural stability, respectively. Following the clinical assessment, participants were asked to complete four barefoot walking trials at a self-selected and comfortable pace along a flat and level 14-m walkway whilst looking straight ahead. The time taken to traverse the central 6-m distance was recorded using a handheld stopwatch; in accordance with the protocol for the 6-Metre Walk Test. Following the completion of the 6-Metre Walk Test trials, participants were asked to complete two modified 6-m Timed Up and Go assessments.

### Outcomes

**Fig. 1:**
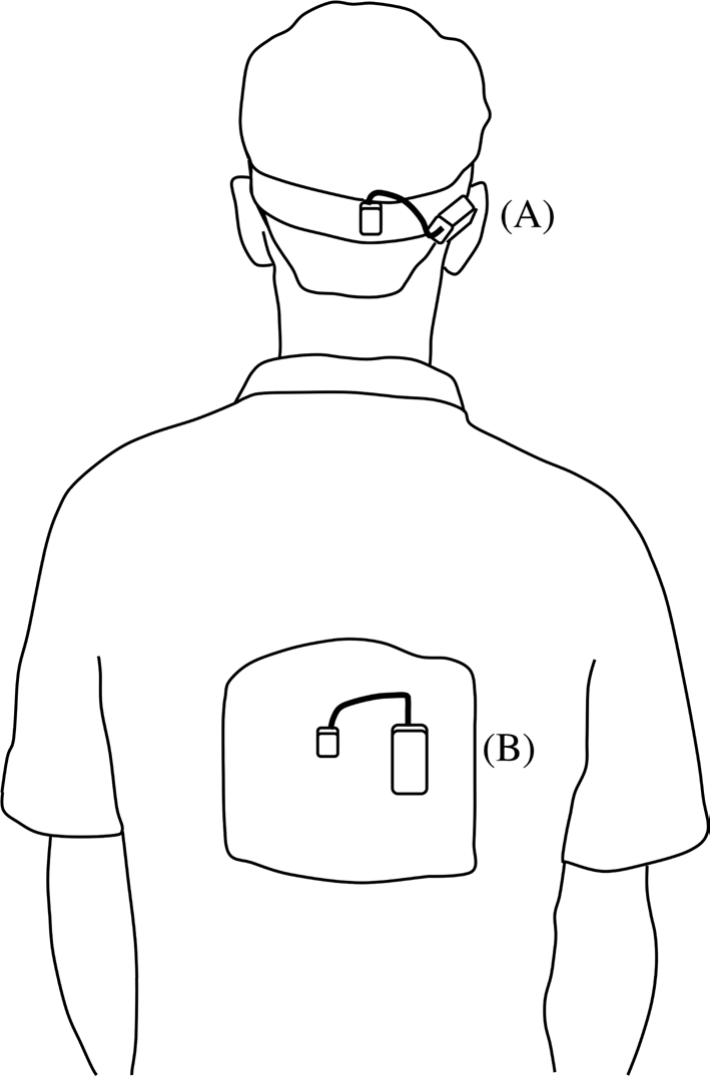
Illustration of tri-axial accelerometers affixed **A** to a headband and **B** to the participant’s back

Commensurate with previous research, tri-axial accelerometers (1500 Hz; Noraxon Inc., Scottsdale, AZ) were firmly affixed to a headband positioned over the occipital protuberance of the skull and directly to the skin overlying the spinous process of the 10th thoracic vertebra using double-sided tape [11, 13, 26] (Fig. 1). During the walking tasks, accelerations were wirelessly telemetered to a Noraxon Telemyo DTS unit connected to a laptop computer running the MyoResearch XP (v1.08) software. Raw accelerations for each trial were subsequently truncated to include 8 continuous gait cycles (i.e. 4 right/4 left) in the middle of the walking trial; yielding 32 individual gait cycles for each participant under each stimulation condition. This approach is consistent with previous research that has assessed harmonic ratios in younger adults [18], older adults [27], and people with clinical conditions, like PD [13, 28] and Multiple Sclerosis [29]. The raw accelerations recorded by the three-dimensional accelerometers included both accelerations relating to movement and gravitational acceleration. To separate the movement-related accelerations from the acceleration due to gravity (constant value of − 9.81 m/s^2^), a previously described and extensively used rotational algorithm was employed [30]. In short, this procedure uses an extension of trigonometry to mathematically rotate (transform) the three-dimensional accelerations collected by the wearable devices to ensure that gravitational acceleration is only represented along their vertical axes. Following this process, it was possible to subtract the gravitational constant and analyse the movement-related accelerations separately. Data were then low-pass filtered using a fourth-order Butterworth filter with a cut-off frequency of 30 Hz and subsequently analysed in the frequency domain using the well-established Fourier series technique [31] with the fundamental frequency of the signal derived from stride duration [32]. Most of the power in walking-related accelerations occurs at or below 10 Hz [19, 33], hence, the harmonic ratio was calculated along the anterior–posterior (AP), medial–lateral (ML), and vertical (VT) axes for the head and trunk by dividing the sum of in-phase harmonics by the sum of out-of-phase harmonics using the first 20 harmonic coefficients [19, 30] (Additional file 1: Figure S1). Higher harmonic ratios represented more in-phase harmonics relative to out-of-phase harmonics and, hence were considered to represent greater gait rhythmicity and dynamic postural stability [19]. From the recorded acceleration signals, the root mean square (RMS) amplitude of the time-series data was also calculated to provide insight into the magnitude of head and trunk accelerations in the AP, ML, and VT directions [11]. The RMS amplitude of the segmental accelerations provided insight into the magnitude of movement exhibited by the head and trunk during the walking tasks.

Trunk accelerations were also used to derive several temporal gait measures, by identifying the timing of foot contacts using peak vertical trunk accelerations [11]. By summing the number of steps taken by each participant during each walking trial and dividing this by the time taken in minutes, it was possible to determine cadence (steps/min). Similarly, by determining the time that had elapsed between two successive steps, it was possible to calculate the average step time for each participant (seconds) and step timing variability (standard deviation of the step times, recorded in milliseconds). All accelerometer-based analyses were performed using a custom developed MATLAB program (v7.13, The MathWorks, USA).

Individual DBS electrodes were identified by merging the postoperative CT scans with the preoperative MRI using 3D Slicer v4.11 to manually mark the mid-point appearing as hyperintense voxels due to metallic artefact [34]. Images were aligned along the Anterior and Posterior Commissures to normalize brain orientation using acpcdetect v2.0 (NeuroImaging Tools & Resources Collaboratory, https://www.nitrc.org). The three-dimensional coordinates for the ideal neurosurgical target within each STN were determined separately for each hemisphere of the brain by an experienced neurologist [35]. It is worth noting that the marking of both the ideal target in the STN and the DBS electrode is a manual process and thus may introduce issues, such as bias and inter-operator variability. To limit the risk of these potential confounds, these processes were completed by the same experienced neurologist and experienced data analyst for all the DBS electrodes; consistent with recent work [36]. These data were subsequently used to calculate the distance (in millimetres) between the midpoint of each electrode and the ideal target. The difference between the ideal and actual location of the active electrode was expressed in the form of X (negative = more medial), Y (negative = more posterior) and Z (negative = more inferior) distance, which were combined to provide a Euclidean distance. All distance calculations were performed automatically using a custom script written in Python v3.7 (Python Software Foundation).

### Statistical analysis

Demographic data were reported as aggregate means and standard deviations for the entire group. To examine differences between high- and low-frequency stimulation conditions with respect to the clinical assessments and the accelerometer-based measures of gait, linear mixed model (LMM) analyses with a repeated factor of stimulation (2 levels) were used. Given walking speed has been shown to influence segmental accelerations [27] and was not constrained in this study, it was included as a covariate in each of the LMM analyses. LMM analyses were performed with walking speed and each of the following entered separately as covariates; the Euclidean distance; X distance; Y distance; Z distance; the TEED, and the MDS-UPDRS III. If the model returned a significant main effect or interaction, the Tukey’s Least Significant Difference (LSD) post-hoc test was used to determine where the differences lay. Furthermore, to determine whether the difference between the ideal and actual location of the active electrode significantly influenced the rhythmicity of head and trunk movements, simple linear regression was used. All statistical procedures were conducted using the Statistical Package for the Social Sciences (SPSS) (Version 25, SPSS Inc., USA), with the estimated marginal means and standard errors considered against the *p* < 0.05 level of significance. Following completion of all data analyses, the principal investigator was unblinded to the order of participant testing to allow the study’s outcomes to be appropriately interpreted and discussed. When statistically significant changes were found, the minimal detectable change (MDC) was calculated to confirm whether the changes were clinically meaningful. The MDC score represents the smallest change in an outcome that would reflect a meaningful change in patient function. Hence, this metric provides insight into the clinical importance of the findings.

## Results

### Study population

**Fig. 2:**
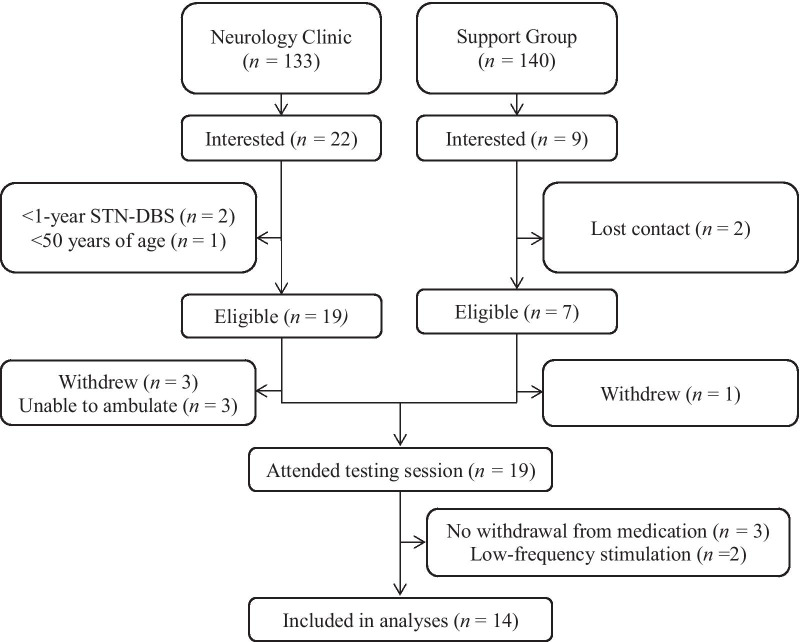
Flow diagram summarizing the recruitment and screening procedures for those invited to participate

Between March and August 2018, 31 post-operative STN-DBS PD patients expressed interest to participate in the study. Of these people, 26 were deemed to be eligible following initial screening and scheduled to attend the data collection session (Fig. 2). Of the 5 participants who were not recruited, 2 were unable to be contacted again after they had made initial contact and 3 were deemed to be ineligible, as their STN-DBS surgery was either < 1 year ago (n = 2) or their age was < 50 years (n = 1). Of the 26 participants recruited into the study, 4 withdrew prior to their scheduled assessment and a further 3 were excluded as they were unable to ambulate following overnight withdrawal from their medication. The remaining 19 participants attended the testing session and completed the objective walking assessments and the clinical assessments for symptom severity. Following data collection, data for 5 participants were excluded due to the participants either reporting that they had taken their anti-Parkinsonian medication on the morning of testing (n = 3) or because their typical (chronic) stimulation settings already involved low-frequency stimulation (n = 2). Data for the remaining 14 participants (Table 1) were included in the subsequent analyses.

### Gait rhythmicity

Linear mixed model analyses that controlled for walking speed, returned a significant main effect for stimulation (low- vs. high-frequency), for the ML and VT rhythmicity of the trunk during the gait trials (Table 2). For each of these components, gait rhythmicity was significantly increased during the low-frequency stimulation condition, compared with the high-frequency stimulation state. Subsequent analyses indicated that these improvements in ML trunk rhythmicity during walking with low-frequency STN-DBS were not significantly influenced by walking speed, Euclidean distance, TEED, or the X, Y, and Z distances of the active electrodes. Further, given the mean difference between the ML trunk rhythmicities observed during the high- and low-frequency conditions (0.42) was greater than the MDC score for this outcome (0.32), this statistically significant improvement was also considered to be clinically meaningful. When controlling for symptom severity, a significant main effect was found for trunk rhythmicity in the AP, ML and VT directions. Simple linear regression analyses showed that, during the high-frequency stimulation condition, having more ventrally located active electrodes was predictive of poorer trunk rhythmicity in the ML (B = 0.19, *p* = 0.030), and VT (B = 0.40, *p* = 0.017) directions. Only the magnitude of VT trunk movement was found to be significantly increased with low-frequency stimulation compared with high-frequency.

**Table 2:** Temporal and accelerometer-based measures of gait for PD patients receiving high-frequency stimulation (HFS) and low-frequency stimulation (LFS) during self-selected and comfortable walking

	HFS (n = 14)	LFS (n = 10)	Speed (1.08 m/s)	Speed (1.09 m/s) and ED (2.31 mm)	Speed (1.09 m/s) and X distance (-0.76)	Speed (1.09 m/s) and Z distance (-0.18)	Speed (1.09 m/s) and Y distance (0.72)	Speed (1.08 m/s) and TEED (92.40)	Speed (1.08 m/s) and MDS-UPDRSIII (31.70)
*Temporal measures*									
Walking Speed (m/s)	1.08 (0.15)	1.08 (0.19)	0.989	0.477	0.471	0.499	0.841	0.400	0.305
Cadence (steps/minute)	126.69 (9.06)	126.66 (11.74)	0.992	0.921	0.816	0.874	0.840	0.840	0.811
Step time (seconds)	0.53 (0.04)	0.53 (0.05)	0.755	0.965	0.885	0.975	0.928	0.928	0.623
Step time variability (ms)	24.5 (14.22)	22.76 (17.26)	0.707	0.825	0.641	0.740	0.671	0.671	0.839
*Harmonic ratios*									
Head AP	1.83 (0.51)	2.04 (0.63)	0.259	0.530	0.523	0.556	0.543	0.308	0.125
Head ML	2.25 (0.54)	2.39 (0.69)	0.161	0.297	0.254	0.264	0.252	0.124	0.075
Head VT	2.57 (0.78)	2.96 (0.80)	**0.048**	0.196	0.182	0.179	0.176	0.143	0.060
Trunk AP	1.78 (0.43)	2.06 (0.44)	0.160	0.189	0.189	0.180	0.184	0.093	**0.040**
Trunk ML	1.81 (0.50)	2.23 (0.61)	**0.004**	**0.012**	**0.011**	**0.010**	**0.011**	**0.010**	**0.002**
Trunk VT	2.9 (1.01)	3.34 (1.11)	**0.044**	0.093	0.068	0.094	0.075	0.080	**0.044**
*Movement amplitude (m/s*^*2*^*)*									
Head AP	1.18 (0.53)	1.20 (0.41)	0.363	0.139	0.135	0.148	0.138	0.336	0.330
Head ML	1.07 (0.23)	1.02 (0.20)	0.782	0.689	0.675	0.683	0.727	0.660	0.522
Head VT	2.07 (0.32)	2.09 (0.46)	**0.034**	0.116	0.134	0.122	0.126	0.083	0.146
Trunk AP	0.91 (0.16)	0.92 (0.21)	0.589	0.757	0.804	0.806	0.812	0.566	0.384
Trunk ML	1.23 (0.35)	1.18 (0.29)	0.131	0.668	0.673	0.675	0.676	0.173	0.255
Trunk VT	2.28 (0.31)	2.33 (0.47)	**0.003**	**0.026**	**0.028**	**0.027**	**0.027**	**0.004**	**0.018**

Data represent the mean (± 1 standard deviation) and p-values derived from the linear mixed model (LMM) analyses. LMM analyses were performed with walking speed and each of the following entered separately as covariates; the Euclidean distance; X distance; Y distance; Z distance; the total electrical energy delivered (TEED), and the MDS-UPDRS III. The values presented in brackets under each of these column headers represent the values used for each covariate within the statistical models

*ns*  no significant differences, *AP*  anterior-posterior, *ED*  Euclidean distance, *HFS*  high-frequency stimulation, *LFS*  low-frequency stimulation, *ms*  milliseconds, *ML*  medial-lateral, *m/s*  metres per second, *VT*  vertical

### Temporal gait outcomes and clinical assessments

There were no significant differences in walking speed, cadence, step time or step time variability between the high- and low-frequency conditions. Furthermore, there were no differences reported for any of the clinical mobility measures (6-m walk or Timed Up and Go test) or symptom severity measures (MDS-UPDRS III or retropulsion test) between the low- and high-frequency stimulation conditions (Table 3). Whilst 5 of the investigated population had reported freezing of gait symptoms, no freezing episodes took place during data collection.

**Table 3:** Clinical measures and stimulation parameters for high-frequency stimulation (HFS) and low-frequency stimulation (LFS) during self-selected comfortable and quick walking speeds

	HFS	LFS	*Sig*
	Mean (SD)	Mean (SD)	
*Clinical measures*			
MDS-UPDRS III	32.7 (10.7)	30.9 (9.8)	0.675
Retropulsion test	1.4 (1.3)	1.1 (1.2)	0.623
Comfortable 6 m walk test (s)	5.3 (0.9)	5.4 (1.2)	0.809
Quick 6 m walk test (s)	4.1 (0.8)	4.1 (0.9)	0.837
Timed up and go (s)	18.9 (5.7)	17.4 (3.4)	0.513
*Stimulation parameters*			
Frequency (Hz)	126.1 (12.3)	60.0 (0.0)	< 0.001
Amplitude (V)	3.3 (0.7)	4.7 (1.1)	< 0.001
Pulse width (μsec)	62.1 (5.5)	62.1 (5.5)	1.000

Data represent the mean (± 1 standard deviation)

*MDS-UPDRS III* motor subscale of the Movement Disorders Society-Sponsored Revision of the Unified Parkinson’s Disease Rating Scale, *s* seconds

Of the 14 participants who completed the assessments, 10 experienced worsening symptoms of resting tremor with low-frequency stimulation. Six of these participants were able to complete the assessments without difficulty, but the remaining 4 were unable to complete the assessments while receiving low-frequency stimulation. Secondary analyses that included only those participants who were able to complete the assessments under both therapeutic conditions confirmed that the reported findings were not biased by the four participants who were unable to complete the low-frequency STN-DBS condition. There was no difference in age, disease duration, time since surgery or electrode location for those who were or were not able to complete the low-frequency STN-DBS condition.

## Discussion

This feasibility study employed a double-blind randomized crossover design to evaluate the effect of low-frequency STN-DBS on objective measures of dynamic postural stability in people with PD. The study’s hypothesis was supported in that we found low-frequency STN-DBS (60 Hz) with a voltage increase to maintain the TEED at the participants’ usual high-frequency stimulation (chronic) level significantly improved gait rhythmicity (higher harmonic ratios) in people with PD compared to high-frequency stimulation. However, it is noteworthy that the low-frequency stimulation strategy was not tolerated by all participants and, in some cases, the gait improvements came at the cost of a re-emergence of tremor.

Rather than investigating the effect of low-frequency stimulation for alleviating freezing of gait, a symptom that is known to respond well to this stimulation strategy [8], this study explored in greater detail its impact on dynamic postural stability during straight line walking. Of the studies investigating different STN-DBS stimulation settings, the outcomes reported for dynamic stability have been almost exclusively based on well-established, albeit largely subjective, clinical scales [9]. While these measures have provided important information regarding the potential efficacy of different STN-DBS settings, objective measures of gait rhythmicity may offer additional and unique insight into the efficacy of such approaches. Considering higher harmonic ratios represent improved gait patterns, the larger values recorded with low-frequency stimulation suggest that this strategy may be effective for improving a patient’s dynamic postural stability. This notion is supported by previous research, which has shown that people with PD exhibit less rhythmic movements (i.e. lower harmonic ratios) than age-matched controls during unconstrained walking [17]. Similarly, in separate research, PD fallers who have not undergone STN-DBS were shown to exhibit significantly poorer head (ML, VT) and trunk (AP, ML, VT) rhythmicities than PD non-fallers [11], which were suggested to reflect reduced dynamic stability in these people. To our knowledge this is the first study to evaluate gait rhythmicity in people with PD following STN-DBS using the accelerometer-based harmonic ratio measure. Our results indicate that low-frequency STN-DBS therapy that is administered with a voltage change to maintain the TEED was effective at improving some aspects of gait rhythmicity in most people with PD following surgery. These findings provide evidence for the potential utility of low-frequency STN-DBS stimulation parameters for post-operative patients who experience gait complications.

Interestingly, the improvements reported in this study were constrained to the medial–lateral and, to a lesser extent, vertical directions, while anterior–posterior rhythmicity was not significantly influenced by the frequency of stimulation. To understand these findings, it may be important to consider what the accelerations measured along each axis of motion (and their subsequent harmonic ratios) represent, with respect to one’s walking patterns. Specifically, during an individual gait cycle, the AP accelerations of the trunk are characterised by two major peaks [19] that depict the attenuated forces resulting from the heel contact made with each step (i.e., one left, one right). To consistently attenuate these forces as they pass upwards through the body, individuals rely on well-timed muscle activations and well-coordinated movements both prior to and immediately following each successive heel strike [37]. Given people with PD exhibit altered gait kinematics [38, 39] and impaired trunk muscle function [40, 41] during self-selected gait, their capacity to consistently attenuate these forces is likely to be impaired. In contrast, ML accelerations of the trunk during locomotion are limb-dependent, and therefore are characterised by a single peak [27], which depicts the shifting of the body’s mass towards the side contralateral to the supporting limb, in preparation for the subsequent step. Given this understanding, our results suggest that low-frequency STN-DBS may improve the rhythmicity (smoothness) with which participants shift their body weight from one foot to the other during locomotion, but have no significant effect on the rhythmicity of the trunk’s movements in the direction of travel. Further research is needed to better understand the mechanism(s) underpinning these reported gait improvements and to further explore the potential limitations of low-frequency STN-DBS for the management of gait dysfunction in people with PD.

In a small number of previous studies, it has been highlighted that some people experience a decline in postural stability with high-frequency STN-DBS, compared with their pre-surgery state [3, 5]. Interestingly, our results showed that participants who have more ventrally located electrodes were more likely to experience deficits in dynamic postural stability during high-frequency stimulation. This finding was complementary to previous research that found ventral stimulation had a significant detrimental effect on temporal-spatial measures compared to stimulation that was focussed more dorsally [42]. High-frequency stimulation at more ventrally located electrodes may have undesired effects on areas immediately inferior to the target, such as the substantia nigra pars reticulata and the pedunculopontine area. Both of these areas are considered to be involved in postural control [43, 44] and are known to respond well to low-frequency stimulation [45–47]. Commensurate with these findings, participants with more ventrally positioned electrodes experience greater improvements in gait and axial symptoms with low-frequency STN-DBS [7].

To date, there have been no double-blind randomised trials to explore the efficacy of low-frequency stimulation for improving dynamic postural stability while statistically accounting for differences in electrode location. The results suggest that, despite a range of active electrode locations, low-frequency stimulation significantly improved gait compared to high-frequency stimulation for the investigated population. Whilst the exact therapeutic mechanism of low-frequency stimulation remains contentious, there is evidence to suggest that the improvements observed with this therapy may be due to the diminished effect it has (compared with high-frequency stimulation) on the neuronal tissues surrounding the STN [48]. The results of the current study suggest that these improvements were independent of electrode location and that other mechanisms may also be responsible. For example, the independent improvement in dynamic postural stability may lend support to a previously identified mechanism, which suggests that a stimulation frequency of 60 Hz, as used in this study, may override the pathological neuronal oscillation in PD and boost the prokinetic gamma band activity [8, 49]. While this increased prokinetic gamma band activity may explain the alleviation of freezing of gait, it is unconfirmed whether this mechanism is responsible for the gait improvements reported in this study with low-frequency STN-DBS. As such, further research is needed to fully understand the influence that variability in active electrode placement has on balance and gait outcomes following STN-DBS in people with PD.

It must be noted that low-frequency stimulation was not tolerated by all participants and, in some cases, the gait improvements came at the cost of reduced therapeutic efficacy for the management of tremor. Specifically, six participants experienced a re-emergence of mild tremor symptoms that did not influence their willingness or ability to complete the assessments, while a further four were unable to complete the assessments due to a re-emergence of this symptom. However, it should be noted that participants were assessed following overnight withdrawal from their anti-Parkinsonian medications to better determine the therapeutic effects of low-frequency stimulation on gait patterns. Therefore, it is possible that the participants’ symptoms of tremor may have been reduced or completely ameliorated with the reintroduction of their pharmacological treatments. A similar re-emergence of tremor was reported in a separate study evaluating the effects of low-frequency STN-DBS [50]; potentially highlighting the need for careful patient selection. Nonetheless, the current study’s findings show that low-frequency stimulation improves dynamic postural stability, regardless of electrode placement. With advances in adaptive DBS technology [51], it may become feasible to deliver low-frequency stimulation to patients for the improvement of dynamic postural stability, while also having a high-frequency stimulation policy to initiate when symptoms of tremor reappear.

Unlike the advanced measures of gait rhythmicity, there were no differences between stimulation conditions for the recorded temporal gait measures. Although these outcomes were commensurate with one previous study [52], they were in contrast to most other research that reported improvements in walking speed with low-frequency STN-DBS [6, 7, 53, 54]. The apparent disparity between the current study’s findings and earlier studies may reflect the largely heterogeneous populations. For example, previous research has found that low-frequency stimulation significantly improved gait speed and reduced step frequency in those who exhibited significant gait disability with high-frequency STN-DBS [53]. Furthermore, the parameters used by different studies when programming the low-frequency STN-DBS adjustment to voltage settings were highly variable, with some making changes to frequency only, while others also made a concomitant adjustment to voltage [9].

Similar to other studies investigating the effect of 60 Hz stimulation on valid, clinically-feasible, although largely subjective assessments of postural stability [50, 52, 53, 55, 56], no significant differences were noted for the retropulsion test between the high- and low-frequency stimulation conditions. Whilst this may be due to the retropulsion test focusing more on stability under static conditions, the similar lack of differences for the clinical mobility assessments seems to suggest that subtle changes in stability and/or gait function are not easily captured with these tools [57]. Therefore, it seems reasonable to suggest that incorporating patient-worn technology into routine clinical practice may provide additional and unique data about gait dysfunction and the efficacy of therapeutic interventions in people with PD [58].

### Limitations

Participants were required to wait a minimum of 60-min before each stimulation condition to allow adequate wash-in time. While it could be argued that a longer wash-in period may have been needed to gauge therapeutic efficacy, the 60-min wash-in/wash-out period was in line with previous studies that have adopted similar methodologies [7, 23, 59]. Nevertheless, the relatively short time period between stimulation conditions means that the results presented in this paper represent the participants’ acute responses. Longitudinal studies are required to determine the long-term efficacy of low-frequency stimulation for STN-DBS PD patients. A second potential limitation is that participants were assessed following overnight withdrawal from their anti-Parkinsonian medications, meaning that, for patients who would usually take medications, the high-frequency stimulation condition would not have been reflective of their best therapeutic state. Nonetheless, similar research involving a non-DBS PD population who presented with primary symptoms of postural instability has shown that, levodopa replacement therapy has varied effects on gait stability and at times, may even be detrimental [28]. Finally, while the randomised crossover design of this study sought to mitigate the risk of learning and/or fatigue biasing the study outcomes, it is important to acknowledge this risk, given data were collected for both conditions on the same day. Where practical, future research should seek to conduct testing across multiple days to better manage the potential risk of patient fatigue and further mitigate the risk of biasing the outcomes.

## Conclusions

This double-blind randomised cross-over study found that low-frequency STN-DBS improved dynamic postural stability in people with PD compared with high-frequency stimulation. Whilst the exact underlying therapeutic mechanism remains unconfirmed, the improvement was independent of the anatomical placement of the active electrode, symptom severity and TEED. Nevertheless, it is noteworthy that low-frequency stimulation was not well tolerated by all participants, as some experienced a re-emergence of resting tremor. For these people, it may be advisable to promote alternate forms of therapy, such as exercise-based interventions [10, 13], to complement high-frequency STN-DBS and improve the patients’ stability. Nonetheless, the results of this study provide evidence for the potential efficacy of low-frequency stimulation for STN-DBS PD patients to improve dynamic postural stability.

## Supplementary Information

**Additional file 1: Figure S1**. Exemplar harmonics of the (i) vertical, (ii) anterior–posterior and (iii) medial–lateral acceleration signal with even harmonics in grey and odd in black and the magnitude as an arbitrary unit normalised to 1.

## Data Availability

The datasets used and/or analysed during the current study are available from the corresponding author on reasonable request.

## Abbreviations

AP: Anterior–posterior
CT: Computed tomography
DBS: Deep brain stimulation
HFS: High frequency stimulation
LFS: Low frequency stimulation
LMM: Linear mixed model
MDC: Minimum detectable change
MDS-UPDRS III: The motor sub-section (Part III) of the Movement Disorders Society-sponsored revision of the Unified Parkinson’s Disease Rating Scale
ML: Medial–lateral
MRI: Magnetic resonance imaging
PD: Parkinson’s disease
RMS: Root mean square
SPSS: Statistical Package for the Social Sciences
STN: Subthalamic nucleus
STN-DBS: Deep brain stimulation of the subthalamic nucleus
TEED: Total electrical energy delivered
VT: Vertical
