# Supplementary material for: Low-frequency STN-DBS provides acute gait improvements in Parkinson’s disease: a double-blinded randomised cross-over feasibility trial
Source: J Neuroeng Rehabil. 2021 Aug 10;18:125. doi: 10.1186/s12984-021-00921-4 (PMC8353795; doi:10.1186/s12984-021-00921-4)
Supplement: Supplementary file 1 — Additional file 1: Figure S1. Exemplar harmonics of the (i) vertical, (ii) anterior–posterior and (iii) medial–lateral acceleration signal with even harmonics in grey and odd in black and the magnitude as an arbitrary unit normalised to 1. [file 12984_2021_921_MOESM1_ESM.docx]

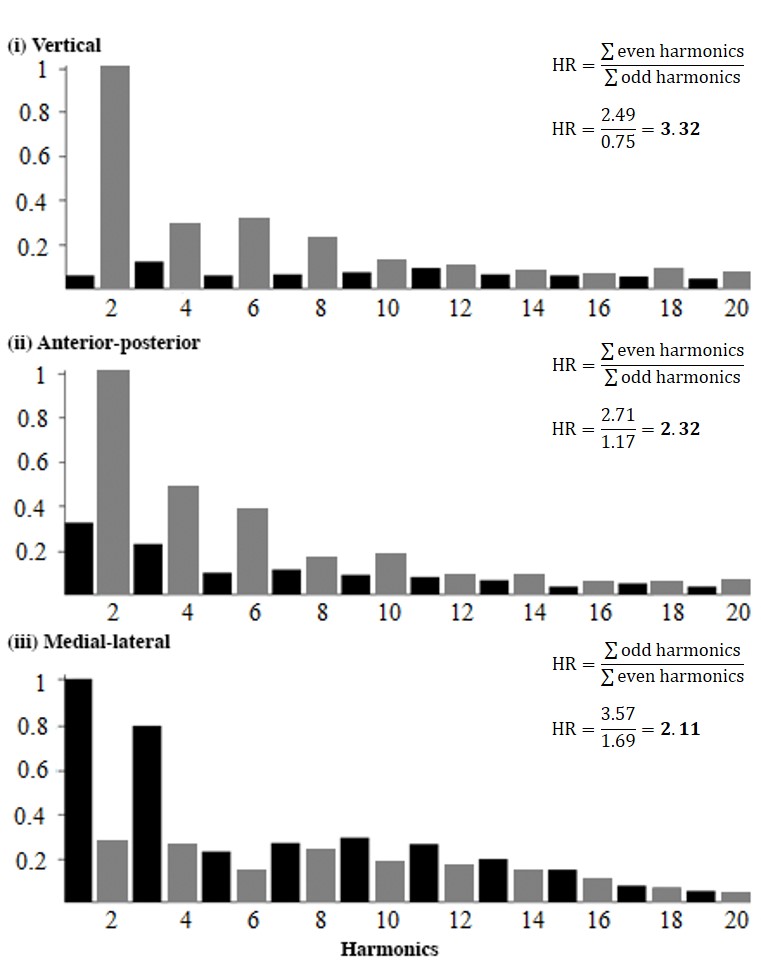


Supplementary material Figure 1: Exemplar harmonics of the (i) vertical, (ii) anterior-posterior and (iii) medial-lateral acceleration signal with even harmonics in grey and odd in black and the magnitude as an arbitrary unit normalised to 1
